# Key Technologies and Evaluation of a MiniSAR Experimental System for Unmanned Underwater Vehicle Detection

**DOI:** 10.3390/s23052490

**Published:** 2023-02-23

**Authors:** Ke Li, Qianqian Liu, Xiang Li

**Affiliations:** 1 Institute of Electronic Engineering, Naval University of Engineering, Wuhan 430033, China; 2 School of Mechanical Engineering and Electronic Information, China University of Geosciences, Wuhan 430074, China

**Keywords:** SAR imaging, submarine detection, Doppler frequency estimation, motion compensation

## Abstract

Synthetic aperture radar (SAR) imaging has important application potential in sea environments research, such as submarine detection. It has become one of the most significant research topics in the current SAR imaging field. In order to promote the development and application of SAR imaging technology, a MiniSAR experiment system is designed and developed, which provides a platform for related technology investigation and verification. A flight experiment is then conducted to detect the movement of an unmanned underwater vehicle (UUV) through the wake, which can be captured by SAR. This paper introduces the basic structure and the performance of the experimental system. The key technologies for Doppler frequency estimation and motion compensation, the implementation of the flight experiment, and the image data processing results are given. The imaging performances are evaluated, and the imaging capabilities of the system are verified. The system provides a good experimental verification platform to construct the follow-up SAR imaging dataset of UUV wake and investigate related digital signal processing algorithms.

## 1. Introduction

Submarines have the characteristics of good concealment and strong raid ability, which are indispensable and extremely important weapons in modern warfare [1,2]. At present, the main method for detecting submarines is based on sonar [3,4,5,6,7,8]. However, with the development of mute technology, the submarines can hardly be detected by sonar. At the same time, due to the multiple reflections of sound waves, the positioning error based on sonar is relatively large. Therefore, simply using traditional acoustic methods cannot meet current requirements of submarine detection. The combination of various submarine detection methods has become a hot research topic.

From current technological development, the method of indirect detection of submarines through radar wakes has great application prospects [9,10,11,12,13]. This is mainly because the radar itself has the characteristics of high precision and strong anti-interference ability, which can ensure the reliability of the detection results. The main idea of this method is to analyze and compare the synthetic aperture radar (SAR) image in the monitoring area with the accurate image class library. Then, the underwater condition can be conjectured, such as whether there is a submarine passing through. The other parameters, such as the diving depth and speed of the submarine, can also be calculated.

Current research shows that the near-field surface waves generated by a submarine’s motion cause a water surface bulge above the submarine, also known as the “Bernoulli hill” [14,15,16]. Therefore, airborne SAR and spaceborne SAR in sea trials can be used to obtain the performance characteristics of submarine wakes under various sea conditions. However, due to military secrecy or other reasons, there are few results of submarine wake detection. In this paper, a small unmanned aerial vehicle (UAV) airborne array interferometric MiniSAR system is designed that can be used for data acquisition and technology validation. The implementation and data processing results of the first flight experiment are presented. The basic results indicate that the experimental MiniSAR system can be a good verification platform for constructing SAR imaging datasets of wake caused by an unmanned underwater vehicle (UUV) and analyzing SAR imaging algorithms. The contents in this paper are arranged as follows: Section 2 describes the configuration of the MiniSAR system; Section 3 explains the flight experiment; Section 4 describes the key image processing technologies in the experiment; Section 5 presents the imaging results, and Section 6 draws the conclusion.

## 2. MiniSAR System

### 2.1. Configuration

**Figure 1:**
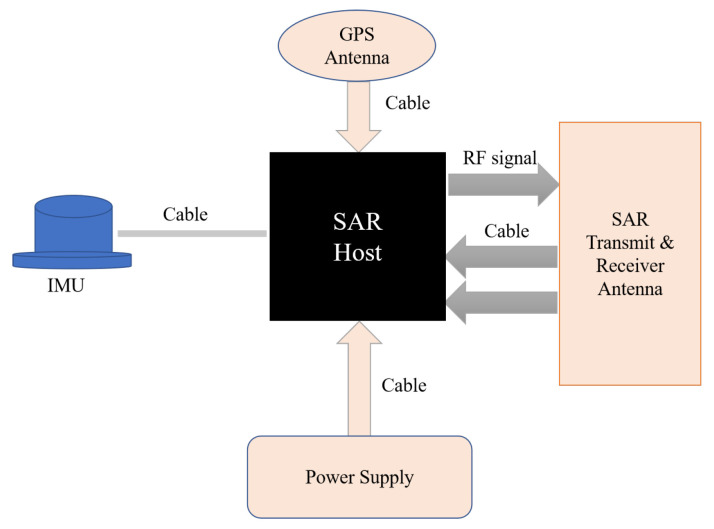
Schematic diagram of the MiniSAR system.

**Figure 2:**
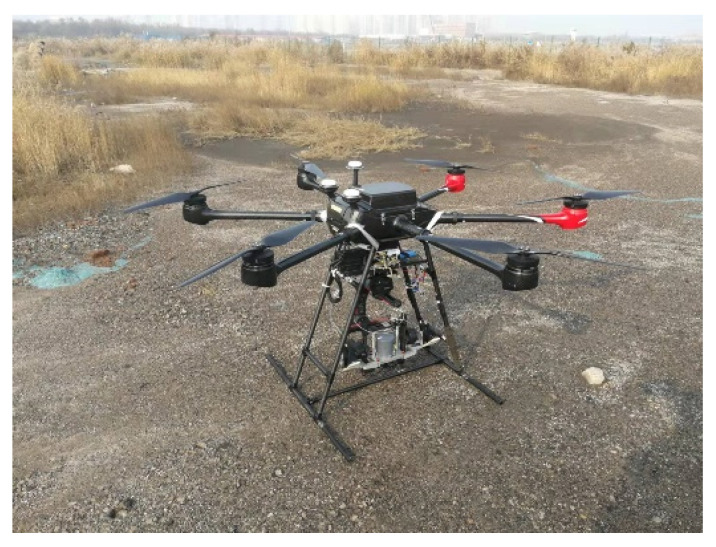
Visible appearance of the MiniSAR hardware.

The MiniSAR system has the characteristics of small size, light weight, and low power consumption and can be installed on platforms such as a UAV [17,18,19]. MiniSAR can actively radiate microwaves to ground targets and receive target echoes. Since microwaves can penetrate rain, clouds, and fog, they can obtain the radar images of various targets in long-distance and large-scale areas in all-day and complex weather conditions. The MiniSAR system is designed and fabricated by Zhongke Yuda (Beijing) Technology Co., Ltd., which includes the SAR host, inertial navigation unit(INU), battery, transceiver antenna, global positioning system antenna, cable, and other related equipment. The SAR host has a 2TB high-speed memory disk, which stores the GPS data, INU data, and original radar echo data. The connection relationship among the systems is shown in Figure 1. It is noted that the SAR antenna is mounted on the UAV platform. Figure 2 shows a visible appearance of the MiniSAR hardware. The main parameters of the UAV are also presented in Table 1. The detailed working of the MiniSAR system will be illustrated in the flight experiment section.

### 2.2. Ku-Band SAR

The core of the MiniSAR system is the Ku-band array interferometric SAR. The main parameters of the Ku-band SAR payload are shown in Table 2. It adopts the frequency-modulated continuous wave with a signal bandwidth of 600 MHz. The center frequency is 14.9 GHz, and the weight is 7.07 kg.

### 2.3. L-Band SAR

Similarly, the main parameters of the L-band SAR payload are shown in Table 3. It also uses the frequency-modulated continuous wave with a signal bandwidth of 400 MHz. The center frequency of the L-band SAR is 1.5 GHz.

### 2.4. Unmanned Underwater Vehicle (UUV)

In this experiment, the Orange Shark III-A is chosen as the unmanned autonomous underwater vehicle. The aircraft has the advantages of large caliber, strong carrying capacity, large diving depth, and wide range of activities. The modular design is adopted to facilitate the needs of different carrying combinations in the future application. The main technical parameters of Orange Shark III-A used in this project are shown in Table 4. A GPS navigator is also installed on the UUV, which can be used to store the trace of the movement of the UUV. The processed data with KML format can be loaded into Google Earth.

## 3. Flight Experiment

**Figure 3:**
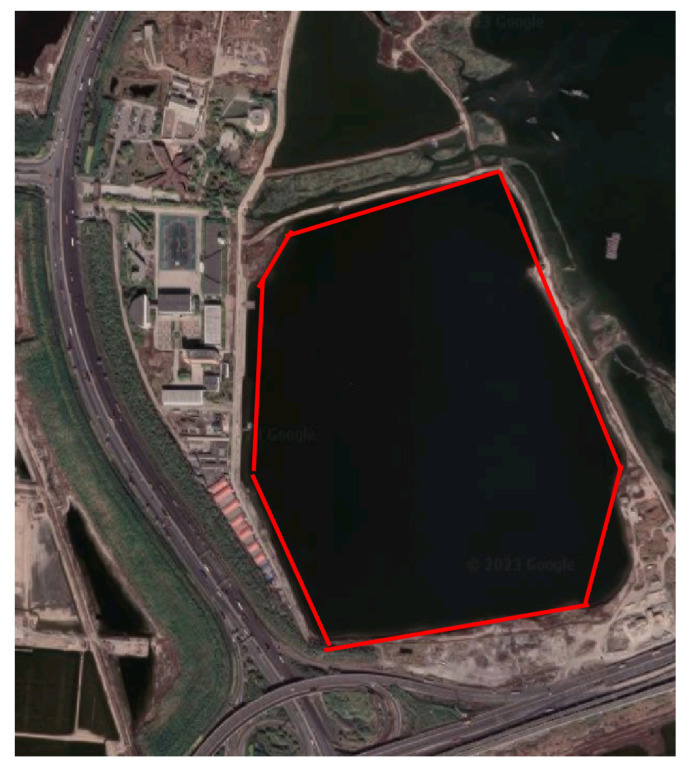
The test area for the detection of wake generated by the UUV.

The test area was selected near the Dagu Fort Museum in Tianjin (the red area of Figure 3). The operational depth of the test water area was 9 m, and the size was 800 m (length) × 640 m (width). The area was selected because the surroundings are relatively empty, which is convenient to lay out the calibrators. The longitude and latitude coordinates of the test area are given in Table 5.

All radar systems were operated in strip mode. The imaging width is related to the flight height, downward viewing angle, and antenna pitch beam width. For Ku-band radar, when the flight altitude is 400 m and the viewing angle is 45 degrees, the near field distance is 186 m, and the far field distance is 857 m. Therefore, the measuring distance is 671 m. For L-band radar, when the flight altitude is 400 m and the viewing angle is 50 degrees, the near field distance is 230 m, and the far field distance is 1092 m. It corresponds to the measuring distance of 862 m.

**Figure 4:**
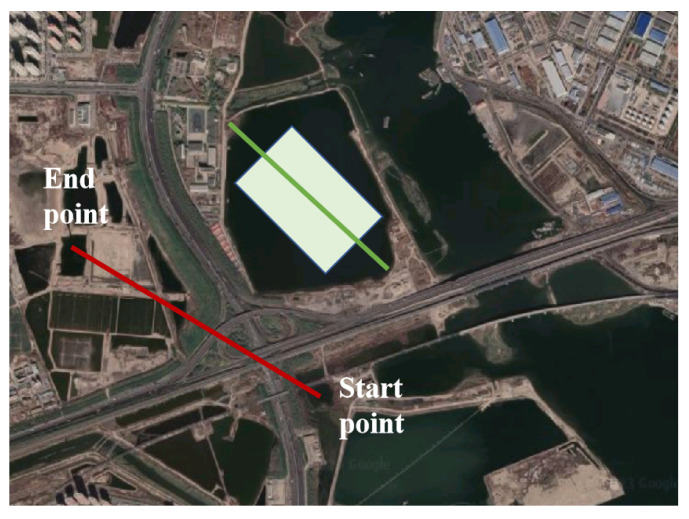
Flight route planning of the UUV and MiniSAR system.

In order to ensure that the imaging center area covered the center of the test water area, the radar route was planned to fly from south to north on the west side of the target water area, from west to east on the north side of the target water area, and from north to south on the east side of the target water area. The route plan is shown in Figure 4.

For the experimental implementation, the UUV was lowered from the shelf to the water area by a large crane. The UUV was wirelessly controlled by the operators from shore side to follow the scheduled route. The parameters of SAR and UVA can be configured from the ground station. Therefore, the status of SAR and UAV were monitored in real-time mode to ensure high-quality detection data.

**Figure 5:**
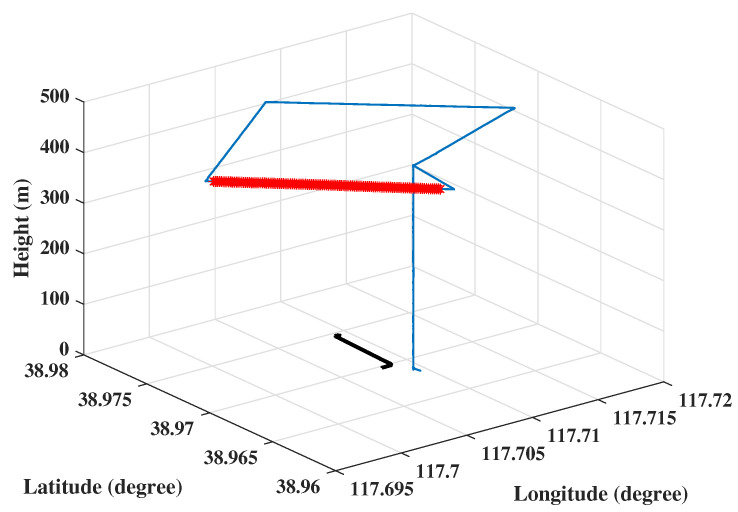
The traces of the MiniSAR system and the UUV.

In order to ensure the wake exists when the MiniSAR system is operating, the MiniSAR system and UUV were launched at the same time. An example of the traces of MiniSAR system and UUV are shown in Figure 5. The IMU in the MiniSAR system is designed for recording the flight path using differential GPS techniques plus acceleration sensors and gyroscopes for each axis. In Figure 5, the blue curve represents the whole process of taking off, climbing, executing the task, and landing of the UAV. The bold red curve represents the trace when the MiniSAR was working, and the black curve represents the movement trail of the UUV.

## 4. Key Image Processing Technologies

**Figure 6:**
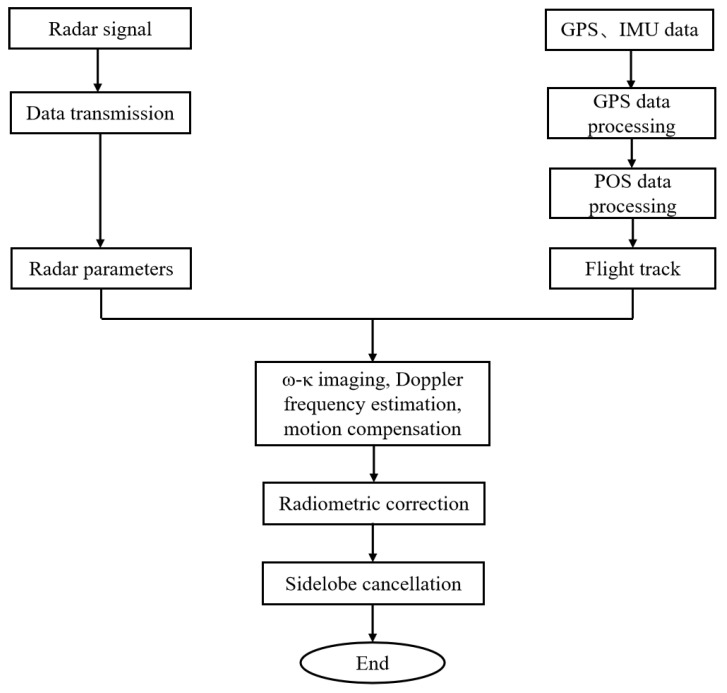
Flowchart of image processing.

The imaging processing steps are shown in Figure 6. The required data include original radar echo data, radar parameters, GPS data, IMU data, route of flight, and data from the position and orientation system (POS). The key steps include ω–κ imaging, Doppler frequency estimation, motion compensation, radio metric correction [20,21,22], and sidelobe cancellation [23,24,25]. The key technologies of ω–κ imaging, Doppler frequency estimation, and motion compensation will be explained in detail below.

### 4.1. ω–κ Algorithm

**Figure 7:**
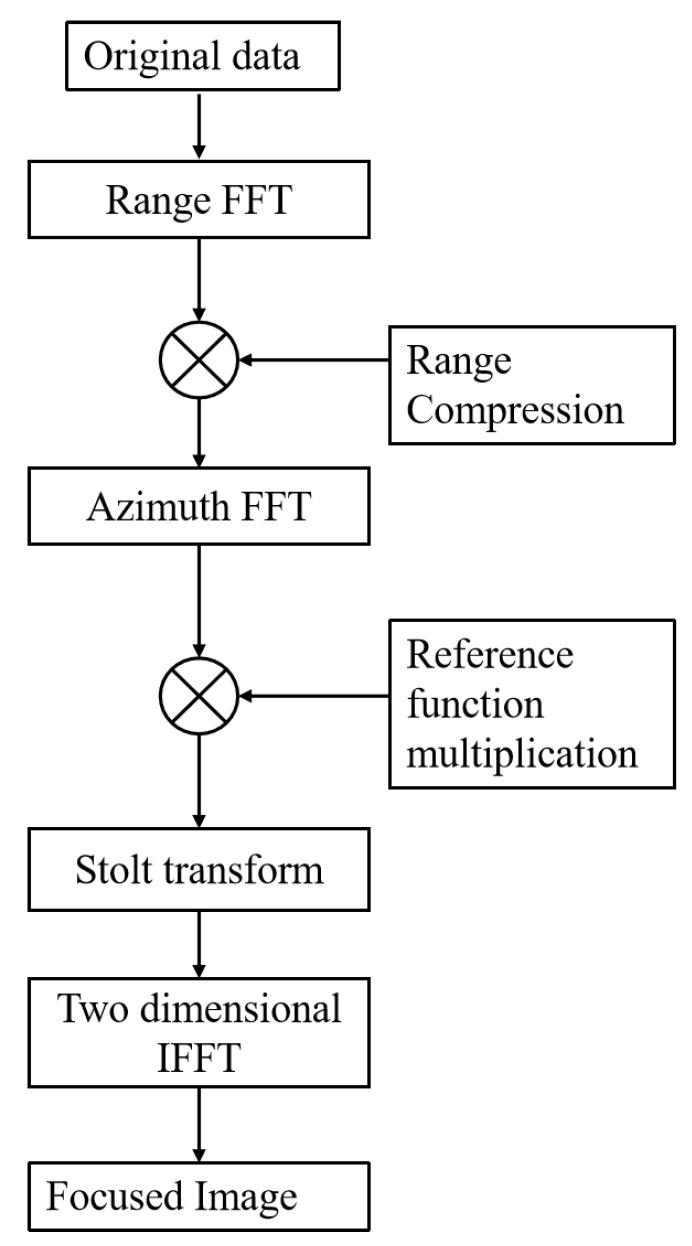
Flowchart of ω–κ imaging algorithm.

This algorithm is a classic algorithm of SAR imaging. The algorithm steps include range compression, reference function multiplication, Stolt interpolation, azimuth compression, and other steps [26,27,28]. The specific algorithm flow is shown in Figure 7.

The key steps of the algorithm lie in the two steps of the multiplication of the reference function and the Stolt transformation. It is noted that when the SAR echo data are collected, there is range migration of the target in the synthetic aperture. Therefore, the two-dimensional frequency domain phase representation of the echo signal is given in Equation (1):

(1) $$\theta_{2df}\left(f_{\tau },f_{\eta }\right)=-\frac{4\pi R}{c}\sqrt{{\left(f_{0}+f_{\tau }\right)}^{2}-\frac{c^{2}f_{\eta }^{2}}{4V_{r}^{2}}}-\frac{\pi f_{\tau }^{2}}{K_{r}}$$

As shown in the above formula, the phase information at different distances is related to both the range frequency $f_{\tau }$ and the azimuth frequency $f_{\eta }$. This shows that the targets at different distances have different migrations.

The reference function multiplication step is to correct all targets of different distances according to the migration value at the reference distance. The phase expression of the reference function at the reference distance is given in Equation (2):

(2) $$\theta_{ref}\left(f_{\tau },f_{\eta }\right)=\frac{4\pi R_{ref}}{c}\sqrt{{\left(f_{0}+f_{\tau }\right)}^{2}-\frac{c^{2}f_{\eta }^{2}}{4V_{r}^{2}}}+\frac{\pi f_{\tau }^{2}}{K_{r}}$$

Multiplying the reference function by the echo signal, the residual phase of the obtained signal in the two-dimensional frequency domain is:

(3) $$\theta_{RFM}\left(f_{\tau },f_{\eta }\right)=-\frac{4\pi (R-R_{ref})}{c}\sqrt{{\left(f_{0}+f_{\tau }\right)}^{2}-\frac{c^{2}f_{\eta }^{2}}{4V_{r}^{2}}}$$

It can be seen from the above formula that at the reference distance $R_{ref}$, the residual phase is zero. It means that the migration has been completely compensated at the reference distance. It is also noted that there is a residual uncompensated phase at the non-reference distance, which needs further compensation.

The Stolt interpolation step can compensate for the residual phase at non-reference distances. Stolt interpolation achieves residual phase compensation by changing the way of signal mapping in the two-dimensional frequency domain. The specific mapping method is given by:

(4) $$\sqrt{{\left(f_{0}+f_{\tau }\right)}^{2}-\frac{c^{2}f_{\eta }^{2}}{4V_{r}^{2}}}\to f_{0}+f_{\tau }^{\prime }$$

Through the above mapping, the phase expression in the two-dimensional frequency domain can be changed to:

(5) $$\theta_{\mathrm{Stolt}\;}\left(f_{\tau },f_{\eta }\right)=-\frac{4\pi \left(R-R_{ref}\right)}{c}\left(f_{0}+f_{\tau }^{\prime }\right)$$

In this way, the phase term in the two-dimensional frequency-domain representation of the signal is linearly related to the new range frequency $f_{\tau }^{\prime }$, which is independent of the azimuth frequency $f_{\eta }$. This means that the Stolt interpolation map completes the residual distance compression by removing the higher-order coupling terms and realizes the migration correction at each distance. Finally, the two-dimensional inverse Fourier transform is conducted to obtain a SAR image with good focusing effect.

### 4.2. Doppler Frequency Estimation

Doppler center frequency estimation is used to estimate the Doppler center of the signal before azimuth compression during the imaging process. Then,the frequency band of the azimuth Doppler signal can be located [29,30,31,32].

There is an “angle-frequency correspondence” relationship in the SAR imaging model. It means that there is a one-to-one correspondence between the azimuth angle and the Doppler frequency of the SAR azimuth signal. The specific corresponding formula is as follows:

(6) $$f_{a}=\frac{2v}{\lambda }\sin\theta$$

where θ is the instantaneous azimuth angle of the echo signal corresponding to the target related to the radar position, and $f_{a}$ is the instantaneous azimuth frequency, that is, the instantaneous Doppler frequency.

When the radar moves in the azimuth direction to form a synthetic aperture, the azimuth angle of the target corresponding to the echo signal changes accordingly, which forms a certain angle interval. This corresponds to a Doppler frequency range, forming a certain Doppler bandwidth. For a SAR system operating in stripe mode, all targets in the scene have a consistent Doppler frequency range within their synthetic aperture. Therefore, the middle value of this Doppler frequency range is the Doppler center frequency, which corresponds to the azimuth of the center of the radar antenna beam.

However, with the change of the flight attitude during the flight of the carrier aircraft, the center azimuth of the radar antenna beam also changes. Therefore, the Doppler center frequency of the radar echo signal is not a constant value. The Doppler center frequency of each piece of data needs to be acquired to form a Doppler center sequence. The commonly used Doppler center estimation methods are roughly divided into two categories. One is to calculate the real-time beam center azimuth of the antenna according to the attitude and position information of the carrier measured by GPS and IMU and then obtain the Doppler center sequence. The other one is to estimate the Doppler center frequency from the collected echo data. The former method is limited by the measurement accuracy of the attitude angle. When the attitude of the carrier aircraft is unstable, the measurement results have a large deviation, resulting in inaccurate Doppler center estimation. The latter method is estimated by the collected echo data, so that the estimated results have high accuracy. In this experiment, an energy balance center frequency estimation algorithm is used, which belongs to the latter method [33].

### 4.3. Motion Compensation Processing

The motion compensation module is used to correct the signal characteristic distortion caused by the motion error of the carrier aircraft. After this operation, the SAR data and the imaging processor can be matched [34,35,36].

For a SAR system with sensors (such as INS, IMU, GPS) that record the data of the operating state of the carrier aircraft, the motion state data of the carrier aircraft can be used to perform motion compensation based on the sensor data. Compared with the motion compensation method based on SAR data, this motion compensation method has more information on the structure of the SAR data because of the use of the real trajectory data of the aircraft flight. Therefore, it can improve the SAR data containing the motion error with more comprehensive motion compensation.

**Figure 8:**
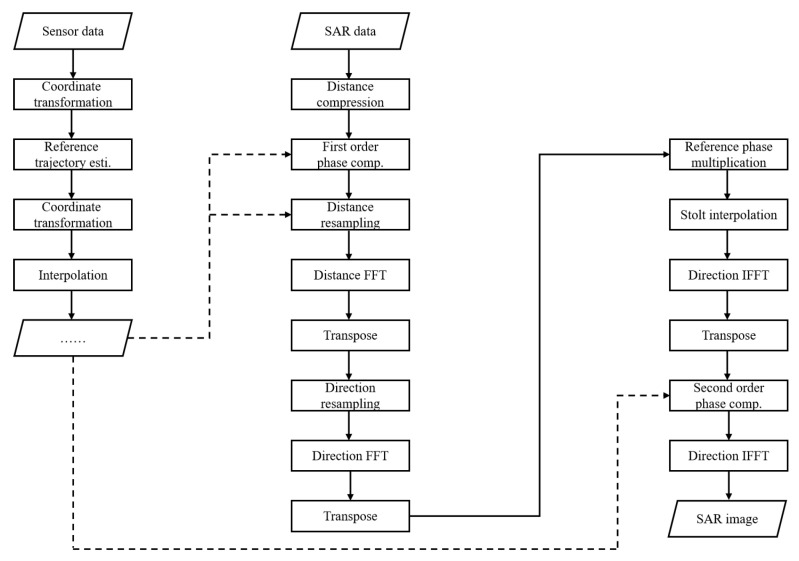
Schematic of motion compensation processing.

The motion compensation module includes range resampling, azimuth resampling, and sensor-based azimuth phase error compensation. The specific flowchart is shown in Figure 8. The implementation is taken in two parts. In the first part, the carrier aircraft state data are allocated to each pulse sampling time by the carrier aircraft trajectory data recorded by the sensor. The sampling frequency of the sensor is usually less than the pulse repetition frequency (PRF), so this step needs to be implemented by interpolation. In this algorithm, the motion sensor needs to provide the longitude, latitude, altitude, and sampling time of the carrier trajectory. In the second part, the motion compensation processing is performed in the ω–κ imaging step using the carrier state data at each pulse sampling time.

## 5. Wake Analysis

The UUV trail is a distributed target, and its probability model is very complex. It is a joint probability density of a surface target, which includes both the random characteristics of the surface SAR signal and the distributed characteristics of the UUV wake signal. The distributed characteristics of UUV wake signals and the random characteristics of surface SAR signals are combined to build a joint probability model of UUV wake SAR signals.

**Figure 9:**
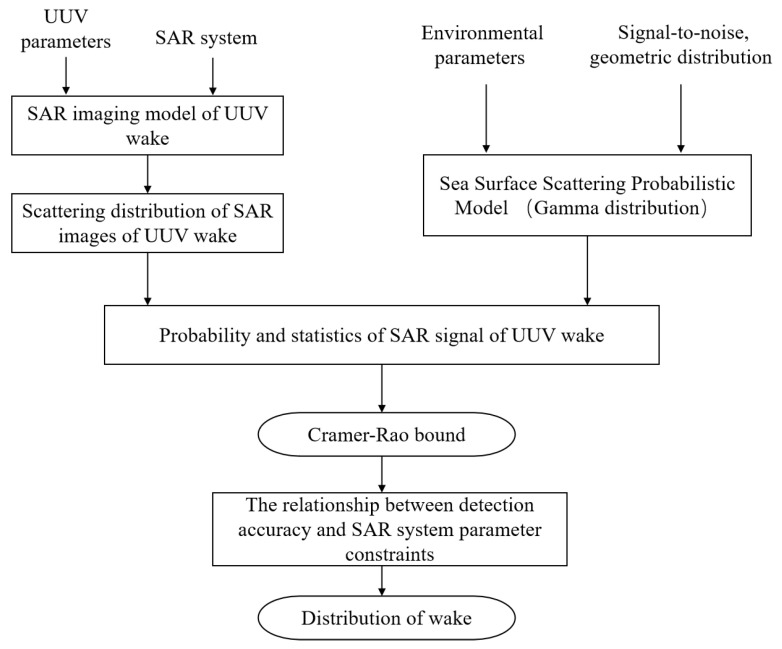
Schematic diagram of processing steps.

When the probability and statistical distribution model is known, the relationship between the detection/estimation accuracy limits of key parameters in the probability model and the key parameters in the probability distribution model can be given according to the Cramer–Rao bound. Based on the method of Cramer–Rao bound, as well as the probability and statistical models of the detection SAR signal, the constraint relationship between the estimation accuracy of key parameters, such as UUV wake intensity, and the SAR system parameters can be obtained. Then, the corresponding data processing technology path is shown in Figure 9.

**Figure 10:**
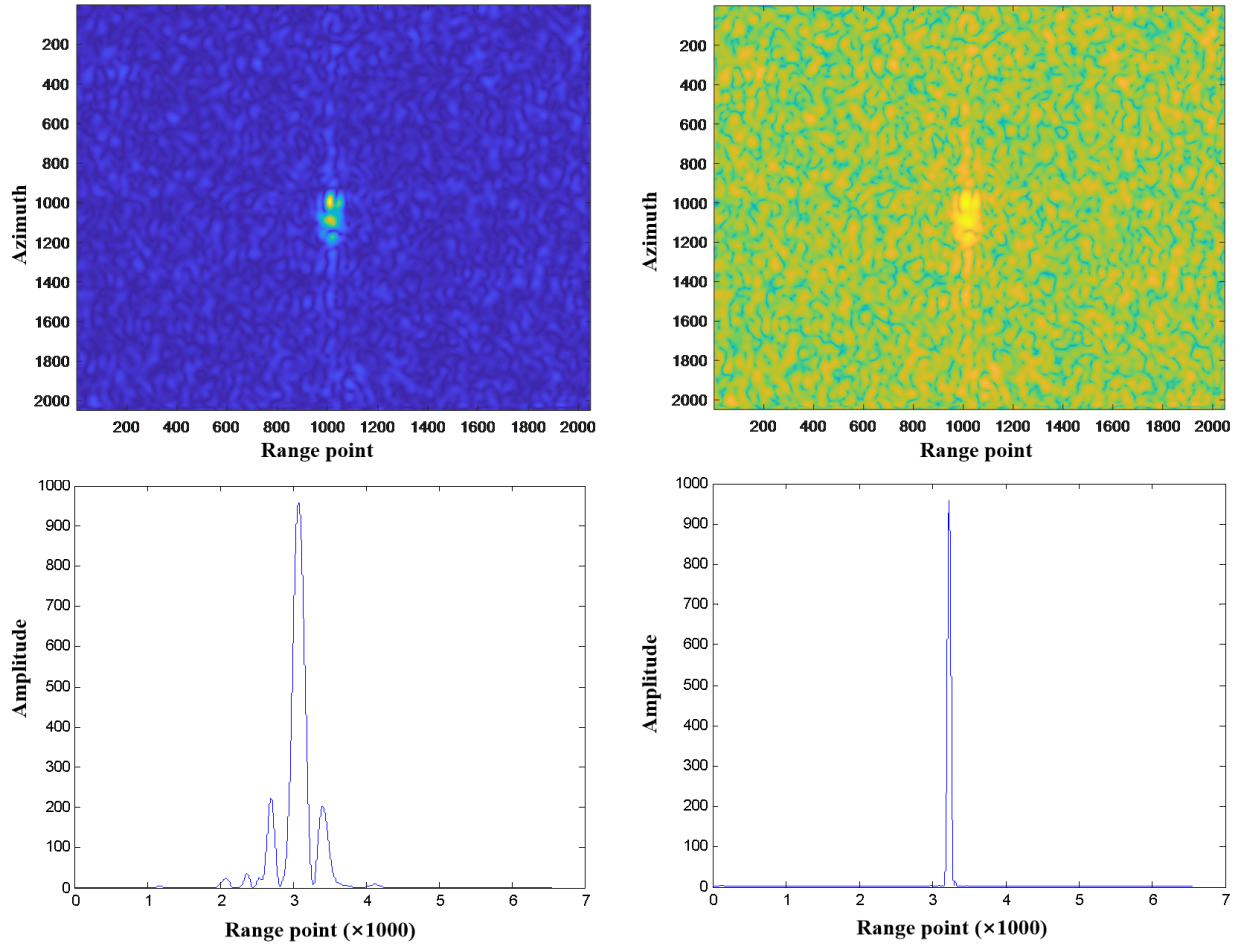
Range and azimuth resolutions of point target in image of L band SAR.

**Figure 11:**
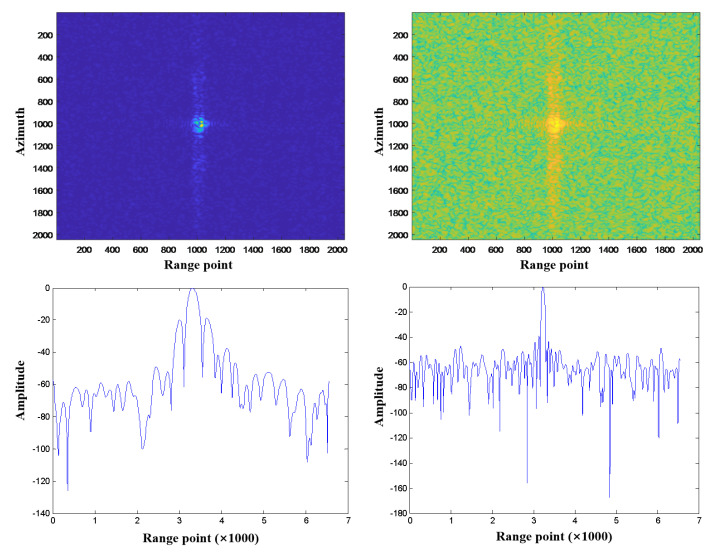
Range and azimuth resolutions of point target in image of Ku band SAR.

In order to check the validity of the MiniSAR system, the information of targeted floating balls in the water is first extracted. Figure 10 and Figure 11 show the imaging results for L-band and Ku-band MiniSAR systems, respectively. Based on the imaging results, the image resolution, peak sidelobe ratio (PSLR), integrated sidelobe ratio (ISLR), and noise equivalent sigma zero (NESZ), are shown in Table 6.

**Figure 12:**
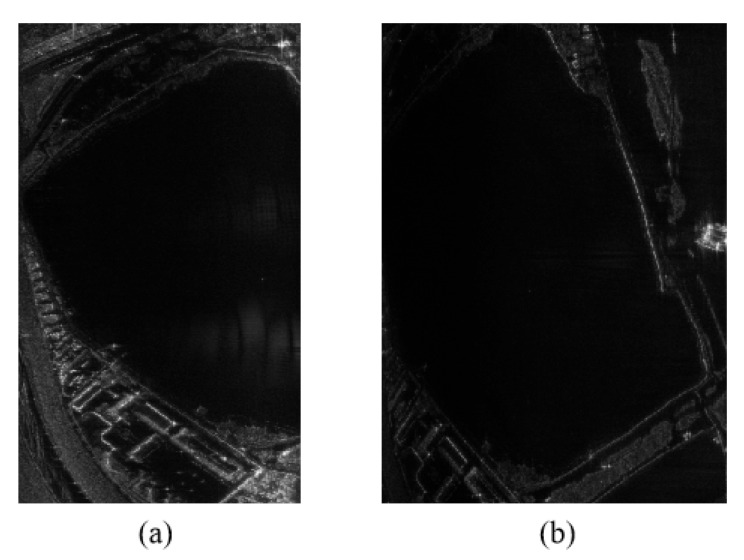
Imaging results of: (**a**) L-band MiniSAR system detection without UUV; (**b**) L-band MiniSAR system detection with UUV having depth of 1 m and speed of 3 knots.

The imaging results for UUV detection based on the L-band MiniSAR system are shown in Figure 12. The imaging results of the L-band MiniSAR system without UUV in the water are presented in Figure 12a. When the UUV is 1 m below the water surface with a speed of 3 knots, the L-band MiniSAR system cannot identify the wake, as shown in Figure 12b. This is mainly because the L-band radar system is susceptible to external signal interference (the signal range of the base station of the operator near the water is 100 MHz–6 GHz), which makes the image polluted by interference and seriously affects the performances of data analysis and processing.

**Figure 13:**
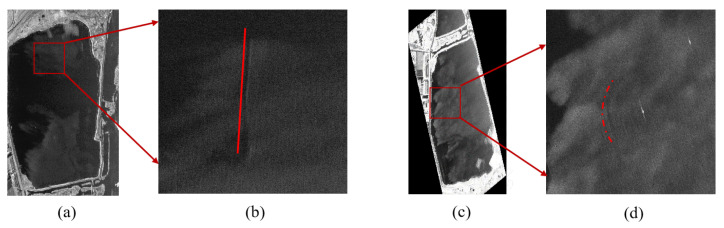
Imaging results of (**a**,**c**) Ku-band MiniSAR system detection with the UUV having a depth of 1 m and speed of 3 knots and (**b**,**d**) enlarged partial figure of (**a**,**c**).

However, under the same experimental condition, the Ku-band MiniSAR system can recognize the wake induced by UUV movement, as shown in Figure 13a,c. The corresponding enlarged partial figure is shown in Figure 13b,d. The difference between the conditions of Figure 13a,c is the movement trail of the UUV.

**Figure 14:**
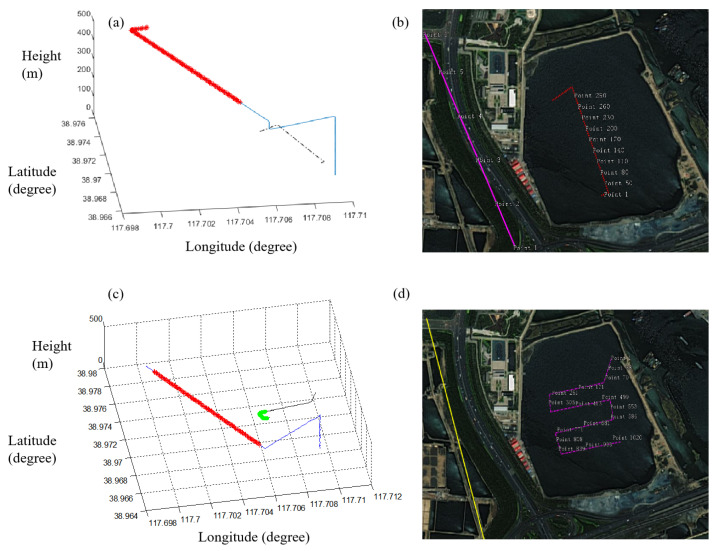
Imaging results of (**a**,**c**) Ku-band MiniSAR system detection with UUV having depth of 1 m and speed of 3 knots and (**b**,**d**) enlarged partial figure of (**a**,**c**).

To verify that the wake is actually generated by the UUV, the movements of the MiniSAR and the UUV are measured in Figure 14. The trajectory data of the MiniSAR and the UUV are first shown in Figure 14a. Then,the movement trail is projected into Google Earth, as shown in Figure 14b. The pink line represents the movement trail of the MiniSAR system (the interval of two points is 10 s). The movement trail of the UUV is represented in the red line (the interval of two points is 1 s). It can be seen that the movement trail of the UUV corresponds to the trail of the wake detected by the MiniSAR system.

A similar case can also be found in Figure 14c, and the green curve represents the detected movement trail. The detailed movement in Figure 14d shows that the UUV turns at point 261, which corresponds to the trail of the wake detected by the MiniSAR system. The reasons that the detected traces are short are possibly because of the short operating time of the MiniSAR under this task and the depth of the UUV may be larger than 1 m in another time.

## 6. Conclusions

In this paper, a MiniSAR experiment operating at L-band and Ku-band to detect the movement of UUV was conducted. The key technologies of Doppler frequency estimation, motion compensation, and image data processing algorithms were described. When the UUV was moving under the depth of 1 m, the imaging results showed that the UUV movement can be identified by the Ku-band MiniSAR system through detecting the wake. The experimental results validate the possibility that the movement of the UUV can be monitored by detecting the wake via a MiniSAR system. Due to the weather conditions and other uncontrollable factors (interference at L band, etc.), the MiniSAR can only detect the wave generated by the UUA under the depth of 1 m. The detection of more movements of UUV under different conditions (depth, speed, etc.) will be left for future work at different locations with smaller interference.

## Figures and Tables

**Table 1:** Key parameters of the UAV platform.

No.	Parameters	Value
1	Motor wheelbase	1765 ± 10 mm
2	Wing span	2530 ± 10 mm
3	Take-off weight without load	22 ± 0.2 kg
4	Maximum load	15 kg
5	Hover time without load	75 min (below 1000 m hight, 25 ${}^{\circ }$C)
6	Hover time with 5 kg load	50 min (below 1000 m hight, 25 ${}^{\circ }$C)
7	Hover time with 10 kg load	30 min (below 1000 m hight, 25 ${}^{\circ }$C)
8	Maximum flight speed	18 m/s
9	Maximum climbing speed	4 m/s
10	Maximum descending speed	3 m/s
11	UAV arm size	1125 × 470 × 455
12	UAV body size	1085 × 690 × 470

**Table 2:** Key parameters of Ku-band SAR.

No.	Parameters	Value
1	Central Frequency	14.9 GHz
2	Signal Bandwidth	600 MHz
3	Transmit Power	5 W
4	Resolution	0.3 m
5	Range Beamwidth	$30^{\circ }$
6	Azimuth Beamwidth	$6^{\circ }$
7	View of Antenna	$45^{\circ }$

**Table 3:** Key parameters of the L-band SAR.

No.	Parameters	Value
1	Central Frequency	1.5 GHz
2	Signal Bandwidth	400 MHz
3	Transmit Power	100 W
4	Resolution	0.5 m
5	Range Beamwidth	$50^{\circ }$
6	Azimuth Beamwidth	$30^{\circ }$
7	View of Antenna	$30^{\circ }$

**Table 4:** Key parameters of the UUV.

No.	Parameters	Value/Description
1	Size	6070 × ϕ680 mm
2	Weight	1350 kg
3	Maximum Load	200 kg
4	Maximum Depth	1000 m
5	Battery Life	30 h
6	Propulsion/Rudder control	Motor propulsion/Power control system
7	Power supply	Lithium battery
8	Communication	High frequency + Satellite + WiFi
9	Autonomous Navigation System	DVL + Inertial navigation + GPS
10	Sensors	Depth gauge, Altimeter, Cabin thermometer, etc.
11	Carryable Load	Multi-beam system, Side scan sonar, Acoustic communication syste, etc.

**Table 5:** Longitude and latitude coordinates of the test area.

Area	Longitude	Latitude
Northwest	117.7050591014	38.9751421878
Northeast	117.7102000790	38.9762790642
Southwest	117.7064748663	38.9689856514
Southeast	117.7127702222	38.9696898620

**Table 6:** Key parameters of SAR image.

Band	Resolution	PSLR	ISLR	NESZ
L	0.48 m × 0.5 m	−19.13 dB	−17.25 dB	−31.37 dB
Ku	0.28 m × 0.25 m	−19.38 dB	−16.37 dB	−30.65 dB

## Data Availability

The dataset used in this research is available upon valid request to any of the authors of this research article.
